# Supplementary material for: Tempests in the troposphere: Mapping the impact of giant storms on Jupiter’s deep atmosphere
Source: Sci Adv. 2025 Mar 28;11(13):eado9779. doi: 10.1126/sciadv.ado9779 (PMC11952092; doi:10.1126/sciadv.ado9779)
Supplement: Supplementary file 1 — Figs. S1 to S5 [file sciadv.ado9779_sm.pdf]

Science Advances
AAAS

Supplementary Materials for

Tempests in the troposphere: Mapping the impact of giant storms on Jupiter's deep atmosphere

Chris Moeckel *et al.*

Corresponding author: Chris Moeckel, chris.moeckel@berkeley.edu

Sci. Adv. **11**, eado9779 (2025)
DOI: 10.1126/sciadv.ado9779

This PDF file includes:

Figs. S1 to S5

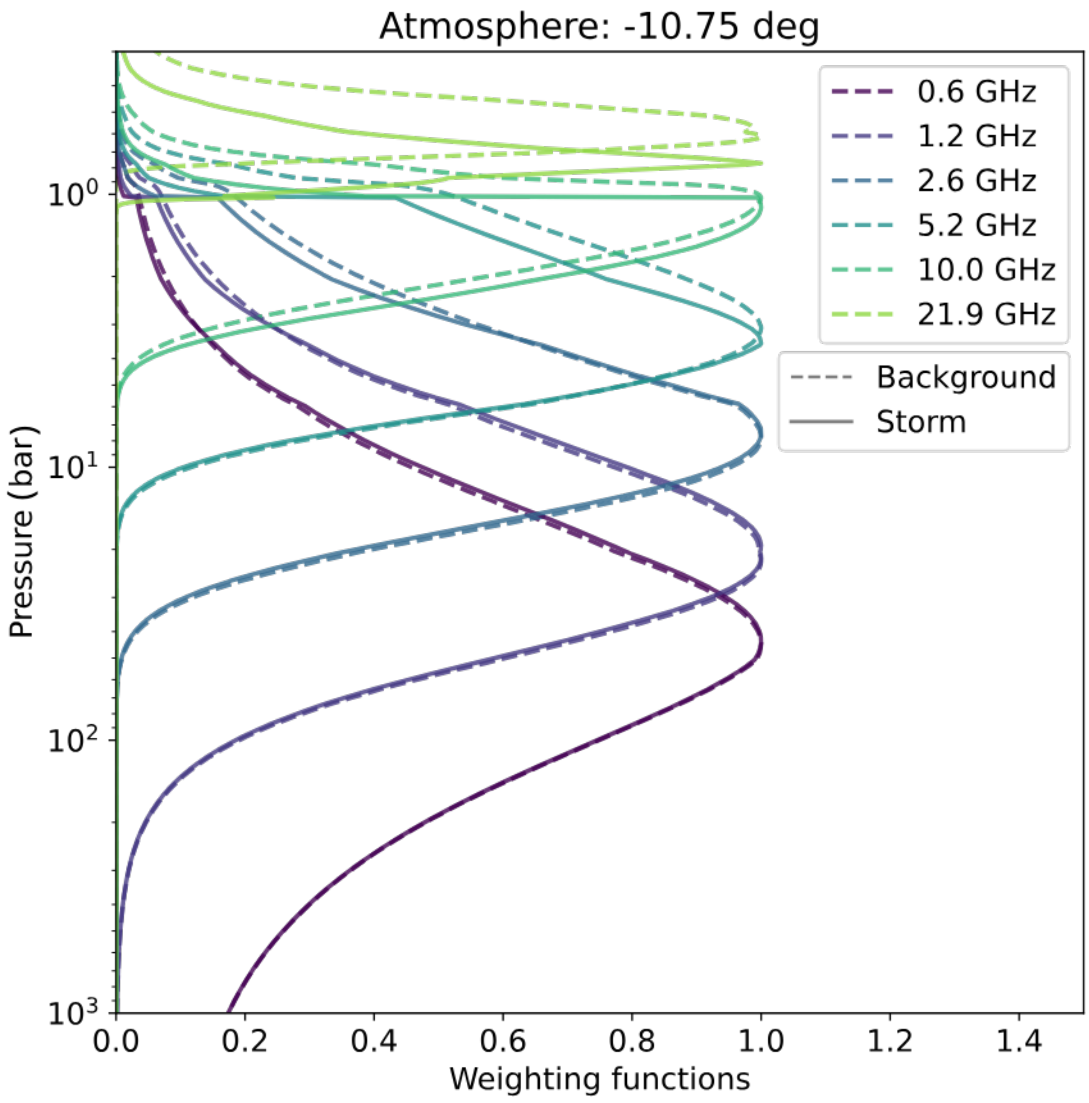

Figure S1: **Weighting functions that represent the depths the emission originates from.** Radio signals record the opacity-weighted thermal emission by the atmosphere. Since the ammonia is the main factor controlling the opacity of the atmosphere, changes in the ammonia abundance will change the pressures from which the thermal signal is received. The dashed lines indicate the pressure from which the thermal emission is received for each frequency based upon the background ammonia profiles. The solid lines represents a storm profile (corresponding to the solid red line in Fig. 3) and the dashed line corresponds to ammonia cloud region (solid blue line in Fig. 3). The depleted upper atmosphere allows us to probe slightly deeper than compared to the background atmosphere.

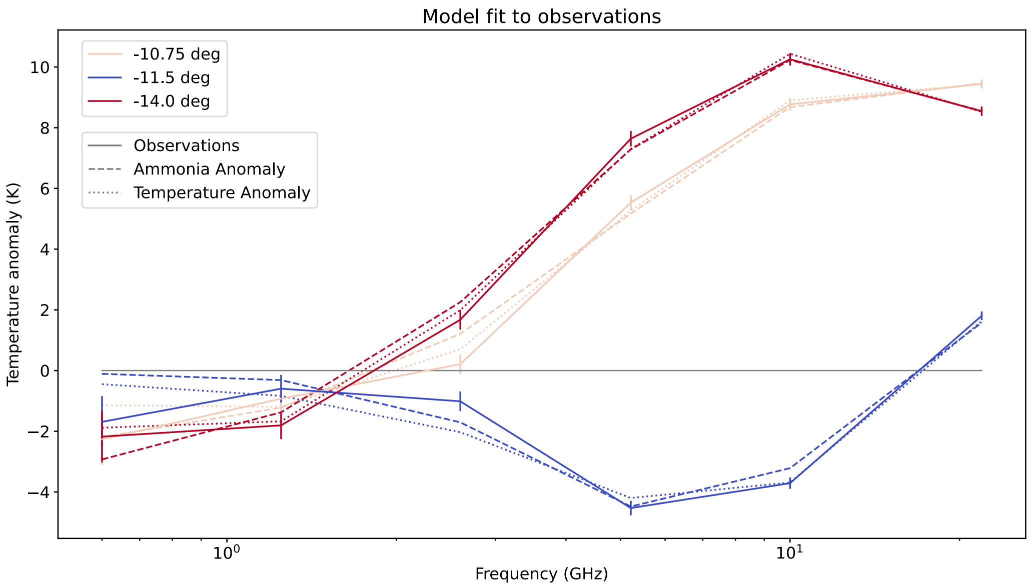

Figure S2: **Comparison of observed and modeled brightness temperature anomaly**. The solid lines represents the observed brightness temperature anomaly for the three regions under consideration in Fig. 3. The dashed line is the modeled brightness temperature difference between the background atmosphere and the storm atmosphere assuming a pure ammonia anomaly. The dotted line shows the result for keeping the ammonia abundance constant and instead showing a pure temperature anomaly. Both models, despite very different assumptions, fit the observations well.

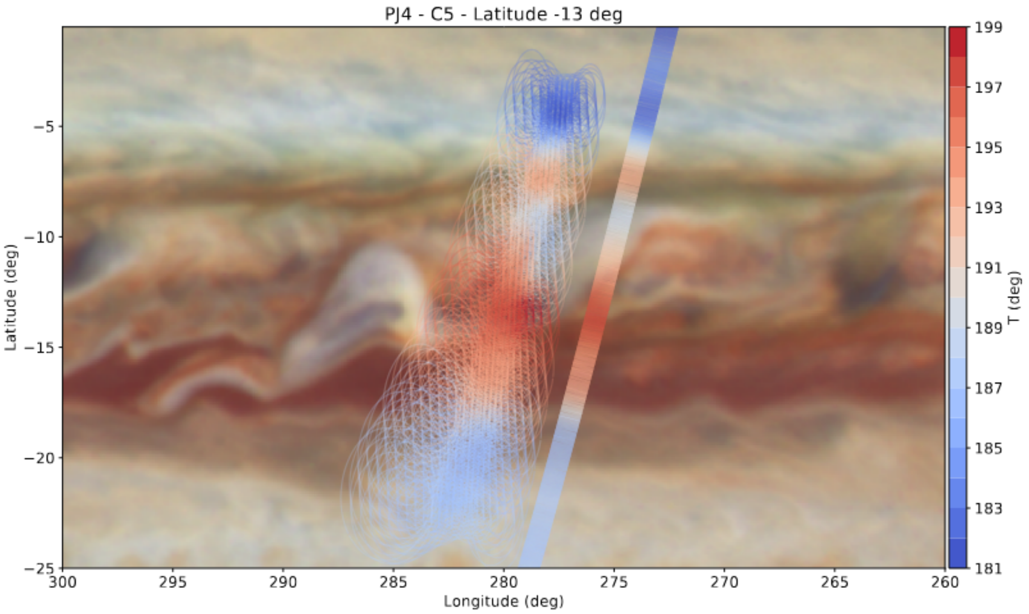

Figure S3: **Example of the distribution of the available individual observations.** Each individual ellipse represents a single integration for the PJ4 flyby in the southern hemisphere. For displaying purposes alone, we have converted each measurement into nadir brightness temperature, by assuming a limb-darkening coefficient. The bar on the right hand side indicates the zonally averaged brightness temperature, and the starting point for the deconvolution algorithm.

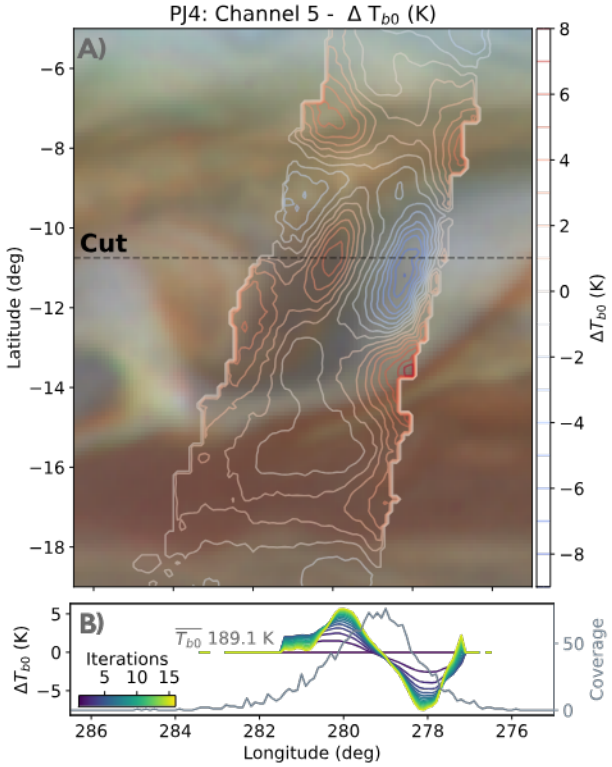

Figure S4: **Example residual distribution for C5 during the PJ4 flyby**. Panel A) shows shows the T_{b0} distribution overlain on concurrent HST observations. The retrieved residuals are largest in regions where the HST observations show a localized white storm plumes next to a darker background. Panel B) is a cross cut through the T_{b0} map where each line corresponds to the summed brightness residual after the number of indicated iterations, and the secondary axis indicates the number of observations for a given region of the map.

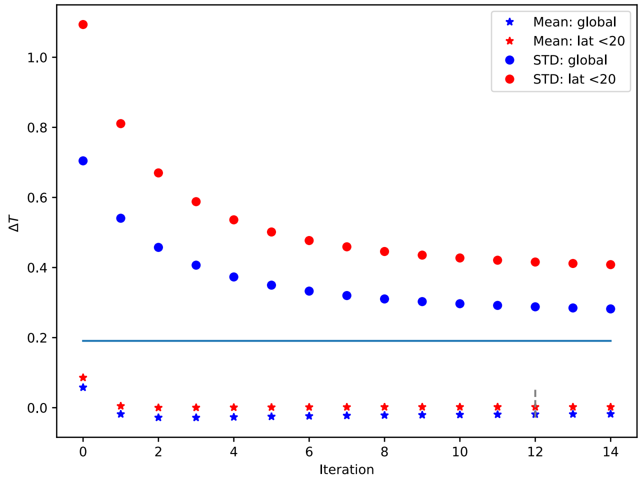

Figure S5: **Convergence of the deconvolution algorithm**. The lines represent the mismatch for PJ4 - C5 across all measurements with the theoretical measurement error approach 0.1 (*14*) indicated by the horizontal line. We can see that within the first few iterations the standard deviations drops quickly until it slowly converges towards the theoretical limit. We track convergence in the tropical regions (lat < 20 deg) and globally, to look for possible contamination by synchrotron radiation.
